# Dual-Specificity Phosphatase 4 Overexpression in Cells Prevents Hypoxia/Reoxygenation-Induced Apoptosis *via* the Upregulation of eNOS

**DOI:** 10.3389/fcvm.2017.00022

**Published:** 2017-04-24

**Authors:** Julie A. Dougherty, Joanna Kilbane Myers, Mahmood Khan, Mark G. Angelos, Chun-An Chen

**Affiliations:** ^1^Department of Emergency Medicine, College of Medicine, The Davis Heart and Lung Research Institute, The Ohio State University, Columbus, OH, USA

**Keywords:** dual-specificity phosphatase 4, reactive oxygen species, mitogen-activated protein kinases, eNOS, Nox4, S-glutathionylation, oxidative stress, cardiovascular disease

## Abstract

Mitogen-activated protein kinases (MAPKs) signaling cascades regulate several cellular functions, including differentiation, proliferation, survival, and apoptosis. The duration and magnitude of phosphorylation of these MAPKs are decisive determinants of their physiological functions. Dual-specificity phosphatases exert kinetic control over these signaling cascades. Previously, we demonstrated that DUSP4^−/−^ hearts sustain a larger infarct and have poor functional recovery, when isolated hearts were subjected to ischemia/reperfusion. Uncontrolled p38 activation and upregulation of Nox4 expression are the main effectors for this functional alteration. Here, dual-specificity phosphatase 4 (DUSP4) overexpression in endothelial cells was used to investigate the role of DUSP4 on the modulation of reactive oxygen species (ROS) generation and vascular function, when cells were subjected to hypoxia/reoxygenation (H/R) insult. Immunostaining with cleaved caspase-3 revealed that DUSP4 overexpression prevents caspase-3 activation and apoptosis after H/R. The beneficial effects occur *via* modulating p38 activity, increased NO bioavailability, and reduced oxidative stress. More importantly, DUSP4 overexpression upregulates eNOS protein expression (1.62 ± 0.33 versus 0.65 ± 0.16) during H/R-induced stress. NO is a critical small molecule involved in regulating vascular tone, vascular growth, platelet aggregation, and modulation of inflammation. The level of NO generation determined using DAF-2 fluorescence demonstrated that DUSP4 overexpression augments NO production and thus improves vascular function. The level of superoxide generated from cells after being subjected to H/R was determined using dihydroethidium-HPLC method. The results suggested that DUSP4 overexpression in cells decreases H/R-induced superoxide generation (1.56 ± 0.14 versus 1.19 ± 0.05) and thus reduces oxidant stress. This also correlates with the reduction in the total protein S-glutathionylation, an indicator of protein oxidation. These results further support our hypothesis that DUSP4 is an antioxidant gene and a key phosphatase in modulating MAPKs, especially p38, during oxidative stress, which regulates ROS generation and eNOS expression and thus protects against oxidant-induced injury or apoptosis. Overall, DUSP4 may serve as an excellent molecular target for the treatment of ischemic heart disease.

## Introduction

Increased reactive oxygen species (ROS) generation and decreased nitric oxide (NO) bioavailability are key contributors to endothelial dysfunction. Many mechanisms have been described contributing to the elevated production of oxygen free radical and endothelial dysfunction. NADPH oxidase, xanthine oxidase, cyclooxygenase, mitochondria, and uncoupled nitric oxide synthase (NOS) are putative sources for ROS generation in the heart and vasculature during ischemia/reperfusion (I/R) injury (1–5). Growing body of evidence suggests that increased oxidative stress alters the function of several enzymes implicated in signal transduction through post-translational modification and protein degradation, including dual-specificity phosphatases (DUSPs) (6, 7). The primary function of DUSPs is to regulate the activities of mitogen-activated protein kinases (MAPKs), thus preventing their overactivation.

Mitogen-activated protein kinases, including ERKs, p38, and JNK, play critical roles in regulating cardiovascular function. The extent of MAPKs phosphorylation and their localization are critical determinants in deciding cell fate: death versus survival (8, 9). Dephosphorylation of these MAPK signal cascades is modulated by MAPK phosphatases or DUSPs. The uncontrolled activation of MAPKs can lead to detrimental effects; therefore, maintenance of proper cell function requires strict regulation of MAPKs activation (10). DUSPs are a subset of the protein tyrosine phosphatases that can inactivate the terminal MAPKs through the dephosphorylation of serine/threonine and tyrosine residues (9, 11). Each of the DUSP members has distinct target specificity (p38, ERKs, or JNK) and subcellular localization. The presence of an active cysteine in their catalytic domain makes DUSPs redox sensitive and susceptible to inactivation by ROS or thiol modification. Dual-specificity phosphatase 4 (DUSP4) is an inducible nuclear phosphatase whose substrates include all three of the MAPKs (ERK, JNK, and p38), and studies have revealed its importance for cardiovascular function (12–19). Overexpression of DUSP4 in human endothelial cells enhances adhesion molecule expression and protects against apoptosis (13). Moreover, DUSP4 gene deletion in mouse embryonic fibroblasts revealed that DUSP4 promotes proliferation and cell survival (20). A more recent study demonstrated that the combined disruption of DUSP1/4 promotes unrestrained p38 activity in both mouse embryonic fibroblasts and in the heart (19). In our earlier study, we demonstrated that *N*-acetyl cysteine (NAC) treatment in endothelial cells upregulates DUSP4 expression through the activation of ERK1/2 (21). In fact, the upregulation of DUSP4 by NAC protects against oxidant-induced cell death and apoptosis *via* the modulation of p38 MAPK activity. These results are consistent with the earlier study in human umbilical vein endothelial cells in which induction by angiopoietin-1 leads to DUSP4 upregulation, which preferentially dephosphorylates ERK1/2, p38, and JNK and delivers antiapoptotic effects during serum deprivation (22).

Furthermore, in our recent study, we have demonstrated that DUSP4 is degraded under hypoxia/reoxygenation (H/R)-induced oxidant stress (18). The degradation of DUSP4 contributes to the overactivation of p38 leading to cell death and apoptosis. Accordingly, cells with DUSP4 gene knockdown and DUSP4 knockout hearts show increased susceptibility to oxidant-induced death and tissue injury. Effectors of this susceptibility include hyperphosphorylation of p38 and upregulation of Nox4 (in DUSP4^−/−^ myocardium) under basal conditions. This study provided the first evidence that DUSP4 is the pivotal phosphatase on the modulation of ROS generation, which subsequently contributes to tissue injury. However, the mechanism and effect of DUSP4 on the modulation of ROS generation after H/R is still unknown. Thus, we further investigate the molecular mechanism of DUSP4 overexpression on the regulation of ROS regeneration during H/R-induced oxidant stress.

Our previous study demonstrated that DUSP4 gene deletion in hearts makes it more susceptible to I/R-induced oxidant stress. An increase in ROS generation and the uncontrolled activation of p38 are the primary contributors for tissue damage after I/R (18). These results agree with our earlier study in endothelial cells with NAC treatment that the upregulation of DUSP4 by NAC protects cells from oxidant-induced cell death and apoptosis by modulating p38 MAPK activity (21). Therefore, in this study, we further investigate the role of DUSP4 in the protection against oxidative stress under H/R using DUSP4 overexpression in endothelial cells. We identified that the beneficial effects of DUSP4 overexpression are *via* modulating p38 activity, increased NO bioavailability, and decreased ROS generation. Therefore, the ability of DUSP4 overexpression simultaneously upregulating eNOS expression and downregulating Nox4 expression in endothelial cells provides a new direction in the treatment of oxidant-induced cardiovascular dysfunction.

## Materials and Methods

### Materials

NOS3 (sc-654) and DUSP4 (sc-1200) antibodies were obtained from Santa Cruz (Santa Cruz, CA, USA); p-ERK1/2 (9101S), ERK1/2 (9102S), p-p38 (4511S), p38 (9212S), GAPDH (2118S), p-JNK (4671S), JNK (3708S), cleaved caspase-3 (9664), and MK2 sampler kit antibodies (9329) from Cell Signaling (Cambridge, MA, USA); GSH (101-A-250) from ViroGen (Watertown, MA, USA). Secondary anti-rabbit (NA934V) and anti-mouse (NXA931) IgG-HRP antibodies were purchased from GE Life Sciences (Piscataway, NJ, USA). RNA was isolated using TRI Reagent^®^ purchased from Ambion (Carlsbad, CA, USA). cDNA was synthesized with a High-Capacity cDNA Reverse Transcription kit from Applied Biosystems (Foster City, CA, USA) and real-time PCR conducted using LightCycler 480 SYBR Green I from Roche (Mannheim, Germany).

### Cell Culture

The maintenance of bovine aortic endothelial cells (BAECs) culture was performed, as previously described by our laboratory (7, 18, 21, 23). For DUSP4 overexpression, cells were seeded to a confluency of 50–60% on six-well dishes and transfected with DUSP4 the next day. Seventy-two hours post-transfection, cells were subjected to H/R treatment. Cells used for immunostaining were grown on sterile glass coverslips coated with attachment factor (Gibco S-006-100, Life technology). First, glass coverslips were coated with attachment factor, and then the excess attachment factor was removed and followed by 30 min incubation in 37°C in the incubator.

### Overexpression of DUSP4 in BAECs

Dual-specificity phosphatase 4 (mouse) overexpression plasmid with CMV promoter was purchased from OriGene (Rockville, MD, USA). For DUSP4 overexpression, cells were seeded to confluency of 50–60% on six-well dishes a day before the transfection. Attractene transfection reagent from Qiagen (Hilden, Germany) was used for DUSP4 transfection. Twelve hours post-transfection, medium was removed and replenished with fresh medium to prevent cytotoxicity from the transfection reagents (7). At 72 h post-transfection, cells were then subjected to H/R treatment to investigate the molecular mechanism of DUSP4 on the modulation of H/R-induced oxidant stress.

### H/R Treatment

Seventy-two hours after DUSP4 transfection, the cell media was removed, cells were gently washed with phenol red-free Dulbecco’s Modified Eagle Medium (DMEM), and fresh phenol red-free DMEM was added to the cells. BAECs were then either incubated in normoxic conditions or placed in a hypoxia chamber (Billups-Rothenberg, CA, USA). The hypoxic chamber was flushed with argon for 30 min (24, 25), and the cells were incubated at 37°C under the hypoxic environment for 1 h. After hypoxia, cells were incubated at normoxic (21% O_2_) conditions for 30 min. After H/R, cells were collected and processed for protein analysis *via* SDS-PAGE and immunoblotting and mRNA quantification *via* qPCR. Cells exposed to only normoxic conditions served as controls and were compared to those subjected to H/R (18, 21).

### Immunostaining of Cleaved Caspase-3 and DUSP4

Bovine aortic endothelial cells were cultured on sterile glass coverslips coated with attachment factor to an 80–90% confluence or 72 h post-transfection. Cells were subjected to H/R treatment (1 h hypoxia and 30 min reoxygenation), cell media was removed, and coverslips were washed 2× with PBS, fixed for 20 min at RT in 4% paraformaldehyde and subsequently washed 3× with PBS containing 0.1% BSA. Cells were then blocked for 45 min at RT in PBS containing 5% BSA and 0.3% TritonX-100 and then incubated overnight at 4°C in anti-cleaved caspase-3 or anti-DUSP4 diluted 1/400 in PBS with 1% BSA and 0.3% TritonX-100. After overnight incubation, coverslips were rinsed 3× with PBS containing 0.1% BSA, and incubated with Alexa Fluor 488 secondary antibody (green) for cleaved caspase-3 and Alexa Fluor 594 secondary antibody (red) for DUSP4 (Cell Signaling, Cambrdige, MA, USA) for 1 h at RT. Finally, coverslips were washed 3× with PBS containing 0.1% BSA, mounted onto a glass slide with ProLong Gold antifade reagent with DAPI (Life Technologies, Grand Island, NY, USA), and fluorescent images (40×) obtained using an Olympus FluoView FV1000 confocal microscope, and data were captured digitally and analyzed (18, 21).

### Immunoblotting

The procedure for the immunoblotting was followed, as previously described (18, 21, 23, 26). Samples were first separated on an 8% Tris-glycine polyacrylamide gel and then electrophoretically transferred to a nitrocellulose membrane. The extent of p38 phosphorylation was determined using the ratio of p-p38 to total p38. Similarly, the extent of ERK1/2 or JNK phosphorylation was determined using the ratio of p-ERK1/2 to total ERK1/2 or p-JNK to total JNK. MK2 phosphorylation at T334 and T222 was used to determine the activity of p38. The extent of eNOS expression was also determined, and GAPDH served as the loading control marker.

### Measurement of Superoxide Generated from BAECs Using Dihydroethidium (DHE) and HLPC Analysis

The level of superoxide generation from control BAECs and BAECs with DUSP4 overexpression or treated with H/R was measured using DHE-HPLC analysis with a slight modification (27). After with or without H/R treatment, cells were washed once with PBS and incubated in Krebs-HEPES buffer. DHE was added to a final concentration of 25 μM and incubated at 37°C for 30 min. The medium was then removed and replenished with fresh Krebs-HEPES buffer for an additional 1 h incubation. After incubation, cells were collected and lysed in 500 μL cold methanol and centrifuged. 2-hydroxyethidium, DHE, and ethidium were separated using a gradient HPLC system (Shimadzu LC-2010A) with a Hypersil Gold column (250 mm × 4.6 mm, ThermoFisher Scientific, Waltham, MA, USA) and detected with a fluorescence detector using an emission wavelength at 580 nm and an excitation at 480 nm. A linear gradient at a flow rate of 0.5 mL/min was developed from mobile phase A (0.1% trifluoroacetic acid) to mobile phase B (acetonitrile) over 23 min from 37 to 47% acetonitrile (18, 21).

### Measurement of NO Generated from BAECs Using DAF-2 and Fluorescent Microscopy

Bovine aortic endothelial cells were cultured on sterile glass coverslips coated with attachment factor to an 80–90% confluence or 72 h post-transfection. Cells were subjected to H/R treatment (1 h hypoxia and 30 min reoxygenation). After with or without H/R treatment, cells were washed once with PBS and incubated in PBS with Ca^2+^ and Mg^2+^. 4,5-Diaminofluorescein diacetate (DAF-2) was added to a final concentration of 5 μM and incubated at 37°C for 60 min (28). After incubation, coverslips were washed once with PBS, mounted onto a glass slide with ProLong Gold antifade reagent with DAPI (Life Technologies, Grand Island, NY, USA), and fluorescent images (20×) obtained using a Zeiss Axiovert 135 microscope.

### RNA Isolation and Quantitative PCR

After transfection with DUSP4 or H/R treatment, cells were first washed with PBS and directly lysed in TRI Reagent^®^. In brief, total RNA was isolated from BAECs *via* the TRI Reaent^®^-chloroform extraction per the manufacturer’s instructions. Extracted RNA was quantified *via* spectrophotometric analysis using absorption spectra at wavelengths of 230, 260, and 280 nm. A total of 1 μg of RNA was reverse transcribed according to the kit’s instructions. Gene expression was detected in triplicate *via* quantitative real-time PCR on a Roche LightCycler480 thermal cycler as follows: 95°C for 10 min (95°C for 10 s, 60°C for 20 s, 72°C for 20 s with signal detection), for 45 cycles. Ct values were calculated by the LightCycler480 Software using the second derivative max method. Data were calculated using the 2^−ΔΔCt^ method (29) and are expressed as target gene transcript fold expression relative to control, following normalization to β-actin (18, 21). Primer sequences are as follows: β-actin forward 5′-TGCCCATCTATGAGGGGTACG-3′, reverse 5′-GGACGATTTCCGCTCGGC-3′; DUSP4 forward 5′-ATTCCGCCGTCATCGTCTAC-3′, reverse 5′-ATAGCCACCTTTCAGCAGGC-3′; NOS3 forward 5′-TACCAGCCGGGGGACCACATAGGC-3′, reverse 5′-CTCCAGCTGCTCCACAGCCACAGAC-3′; NOX4 forward 5′-CAGGGGTCTGCATGGTACTG-3′, reverse 5′-CAGCAGCCCTCCTGATACAT-3′. All primers detected bovine targets, while DUSP4 primers also detected mouse targets.

### Statistical Analysis

Results were expressed as mean ± SEM, *n* ≥ 3. Statistical significance of difference between results was calculated using two sample *t*-tests with an alpha of 0.05. A *P*-value (two-tailed) <0.05 was considered statistically significant.

## Results

### Overexpression of DUSP4 in BAECs Prevents H/R-Induced Apoptosis

**Figure 1:**
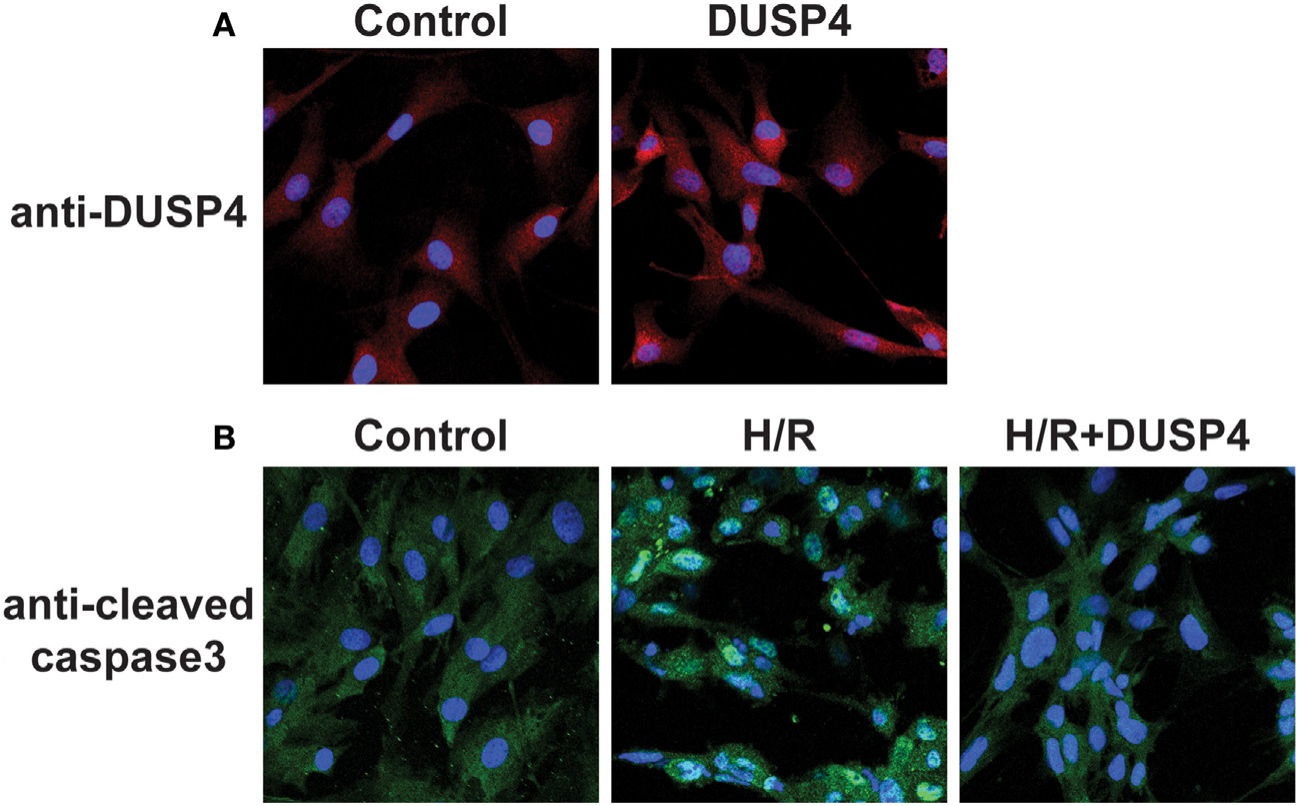
**Dual-specificity phosphatase 4 (DUSP4) overexpression in endothelial cells prevents H/R-induced apoptosis**. **(A)** Bovine aortic endothelial cells (BAECs) immunostaining against DUSP4 (red) and DAPI (blue) staining for nuclei. The population of BAECs overexpressed DUSP4 is 72.67 ± 5.53%, as determined by immunostaining with DUSP4 antibody. The level of DUSP4 overexpression in BAECs is increased by 1.28 ± 0.02-fold compared to control cells (*P* < 0.05, *n* = 3). The result indicated that DUSP4 is overexpressed 72 h post-transfection. **(B)** BAECs immunostaining against cleaved caspase-3 (green) and DAPI (blue) staining for nuclei. Under H/R stress, the percentage of apoptotic cells is increased by 41.08 ± 9.87% compared to the control and DUSP4 H/R groups (*P* < 0.05, *n* ≥ 3). Overexpression of DUSP4 decreases H/R-induced cleaved caspase-3 activation to the similar level of the control and prevents apoptosis (*n* ≥ 3).

Cells were seeded in six-well dishes or on sterile glass coverslips coated with attachment factor to a confluency of 50–60% and transfected with DUSP4 expression plasmids. After 72 h of DUSP4 transfection, cells were then placed inside a modular incubator chamber (Billups-Rothenberg, Inc., Del Mar, CA, USA) and subjected to 1 h of hypoxia. Upon completion of the hypoxic period, cells were reoxygenated at normoxia for a total of 30 min and were subsequently processed for either imaging, immunohistochemistry, or protein analysis. From immunostaining (Figure 1A), DUSP4 is overexpressed in endothelial cells 72 h post-transfection. The population of BAECs overexpressed DUSP4 is 72.67 ± 5.53%, as determined by immunostaining with DUSP4 antibody (Figure 1A). The level of DUSP4 overexpression in BAECs is increased by 1.28 ± 0.02-fold compared to control cells (*P* < 0.05, *n* = 3). Under H/R treatment, cleaved caspase-3 was activated in endothelial cells contributing to apoptosis (Figure 1B). The percentage of apoptotic cells under H/R is increased by 41.08 ± 9.87% compared to the control (*P* < 0.05, *n* ≥ 3). DUSP4 overexpression in endothelial cells suppressed the activation of cleaved caspase-3 to the level of the control and thus prevented apoptosis.

### DUSP4 Overexpression in Endothelial Cells Modulates the Activity of p38 under H/R

**Figure 2:**
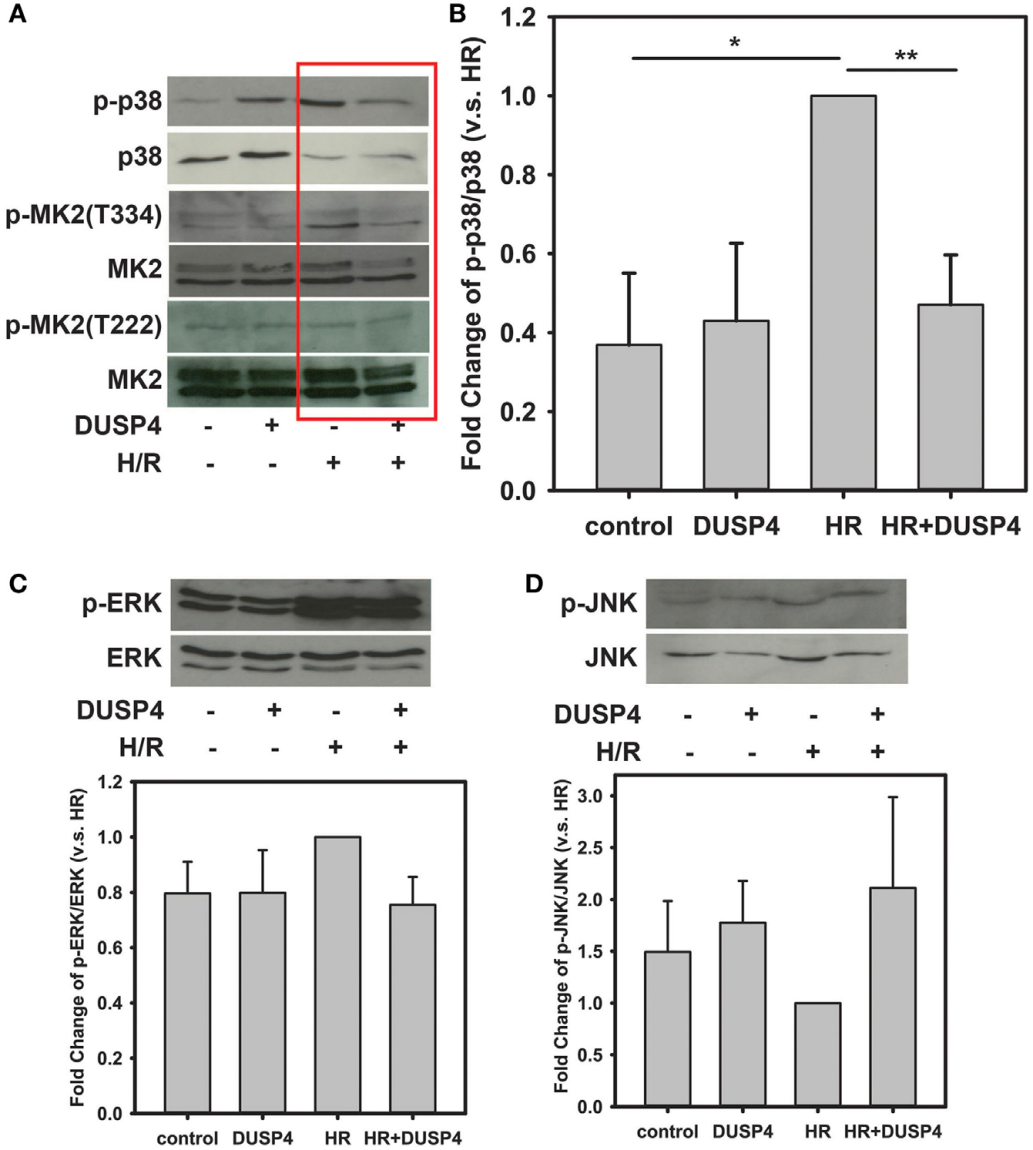
**H/R-induced endothelial cell apoptosis is modulated by dual-specificity phosphatase 4 (DUSP4) and p38 activity**. **(A)** Immunoblotting of bovine aortic endothelial cells after H/R. H/R treatment induces p38 activation. Phosphorylation of p38 subsequently activates its downstream target, MK2 increasing the phosphorylation at the T334 site, which can trigger caspase-3 activation and apoptosis. There was no effect on the phosphorylation MK2 at T222. Overexpression of DUSP4 prevents p38 overactivation and thus provides a beneficial effect for cell survival. **(B)** Statistic analysis of H/R-induced p38 activation. The ratio of p-p38/p38 is an indicator of the activity of p38. The ratio of p-p38/p38 of H/R treated group is set to 1. Overexpression of DUSP4 decreases p38 activity and prevents it from overactivation. *P < 0.01 and **P < 0.005, *n* ≥ 3. **(C)** Under H/R, phosphorylation of ERK1/2 is increased; however, overexpression of DUSP4 does not reduce H/R-induced phosphorylation of ERK1/2. The ratio of p-ERK/ERK of H/R treated group is set to 1. **(D)** There is no effect on the phosphorylation of JNK either under H/R treatment or overexpression of DUSP4. The ratio of p-JNK/JNK of H/R treated group is set to 1. Data are expressed as mean ± SEM, *n* ≥ 3.

p38 is a stress-activated MAPK and plays a critical role in deciding cell fate under oxidative stress. After 1 h hypoxia and 30 min reoxygenation treatment, the phosphorylation of p38 (Figures 2A,B) was over-activated in response to the increased oxidant stress. Overexpression of DUSP4 in endothelial cells prevented p38 overactivation and protected against H/R-induced oxidant stress. The activity of p38 was further determined by measuring the extent phosphorylation of the downstream target of p38, MK2, using immunoblotting. The phosphorylation of MK2 at T334 was dramatically increased consequent to p38 activation (Figures 2A,B). Overexpression of DUSP4 negatively regulated p38 activity under H/R-induced oxidant stress, which further prevented the activation of MK2. There was no effect on the phosphorylation MK2 at T222.

The phosphorylation of ERK was also increased in response to H/R treatment; however, overexpression of DUSP4 did not significantly change the extent of phosphorylation of ERK after H/R (Figure 2C). JNK is another stress-activated MAPK that responds to oxidative stress. There was no significant difference on the phosphorylation of JNK when cells underwent H/R treatment or DUSP4 overexpression (Figure 2D).

### DUSP4 Overexpression Decreases Cellular Oxidative Stress and Superoxide Generation

**Figure 3:**
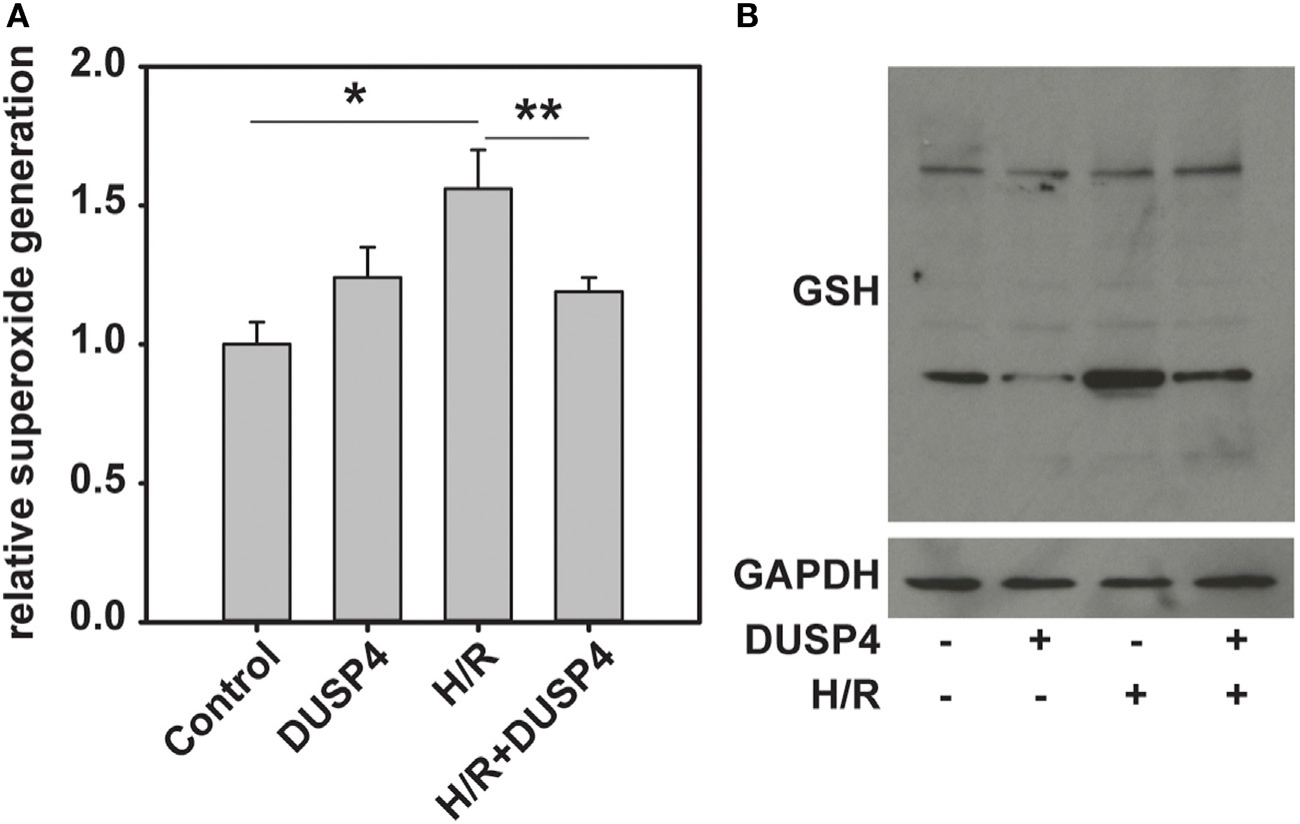
**Dual-specificity phosphatase 4 (DUSP4) overexpression reduces H/R-induced oxidant stress and decreases total protein S-glutathionylation**. **(A)** H/R induces an increase in superoxide generation from cells (1.0 ± 0.08 versus 1.56 ± 0.14). This increase in superoxide generation inhibited by overexpression of DUSP4 (1.56 ± 0.14 versus 1.19 ± 0.05). * and **P < 0.05. Data are expressed as mean ± SEM, *n* ≥ 3. **(B)** The level of total protein S-glutathionylation is an indicator of oxidant stress. Overexpression of DUSP4 deceases H/R-induced protein S-glutathionylation in bovine aortic endothelial cells. Upper panel is the immunoblotting against GSH. Lower panel is the immunoblotting against GAPDH as a loading control.

The level of superoxide generated from cells after being subjected to H/R was determined using DHE-HPLC method. The results indicated that DUSP4 overexpression in cells decreases H/R-induced superoxide generation (1.56 ± 0.14 versus 1.19 ± 0.05, *P < 0.05) (Figure 3A) and thus reduces oxidant stress. The level of total protein S-glutathionylation is an indicator of cellular oxidant stress. The total protein S-glutathionylation was probed using anti-GSH antibody. The results demonstrated that overexpression of DUSP4 decreases cellular ROS generation after H/R treatment and thus decreases total protein S-glutathionylation (Figure 3B).

### Overexpression of DUSP4 Upregulates eNOS Expression and Provides a Beneficial Effect for Cell Survival under H/R

**Figure 4:**
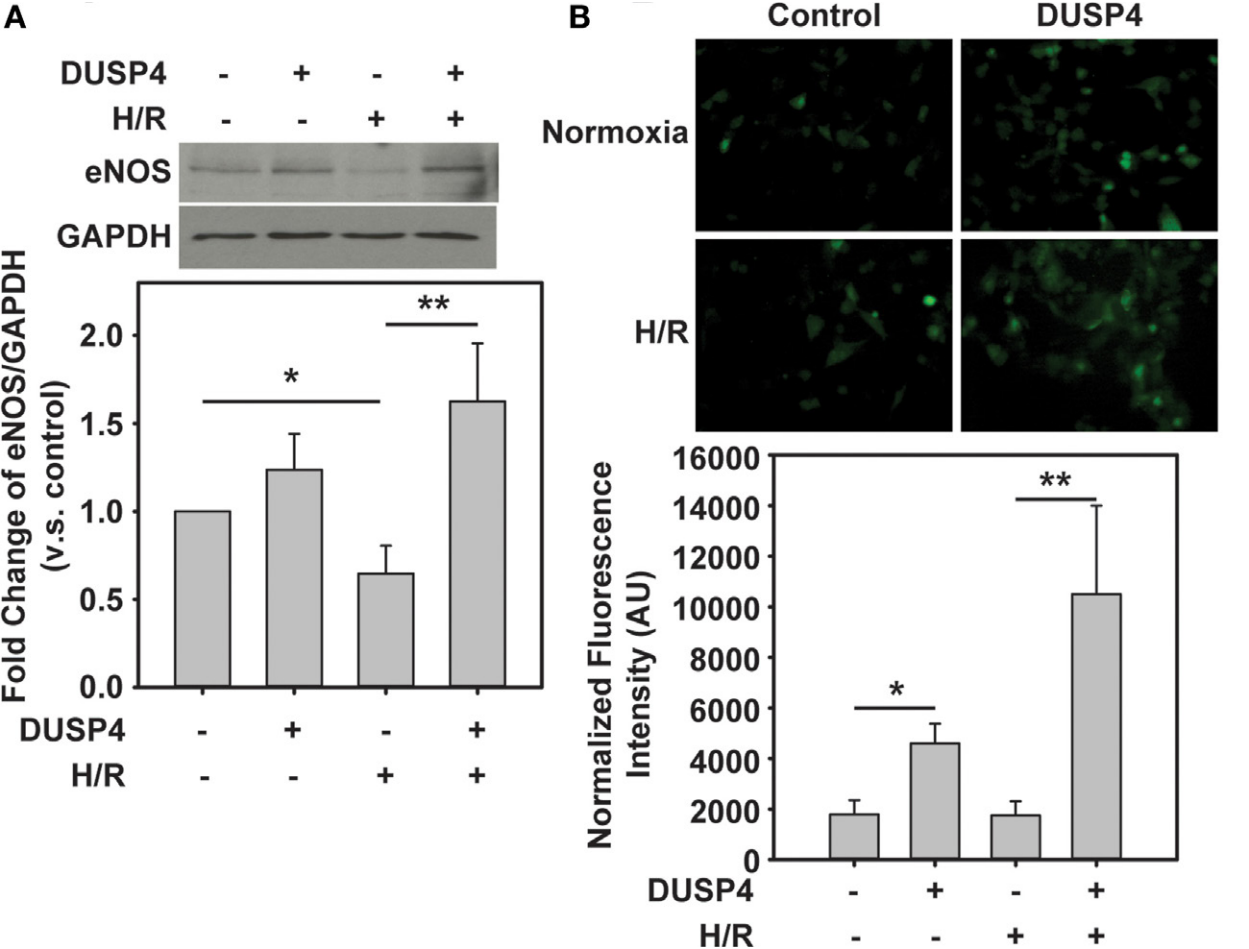
**The beneficial effect of dual-specificity phosphatase 4 (DUSP4) overexpression *via* the upregulation of eNOS**. **(A)** Increased oxidant stress by H/R treatment contributes to eNOS degradation. DUSP4 overexpression upregulates eNOS expression and prevents it from H/R-induced protein degradation. Upper panel is the immunoblotting of eNOS. Lower panel is the immunoblotting of GAPDH as a loading control. **(B)** NO generation from bovine aortic endothelial cells was measured using DAF-2 and fluorescence microscopy in green. DUSP4 overexpressed in endothelial cells not only upregulates eNOS protein expression but also enhances NO generation. The increase in NO generation provides a beneficial effect for cell survival against H/R-induced oxidant stress. * and **P < 0.05. Data are expressed as mean ± SEM, *n* ≥ 3.

eNOS is an important enzyme in endothelium that is responsible for the production of NO. It is a critical small molecule involved in regulating vascular tone, vascular growth, platelet aggregation, and modulation of inflammation. Under H/R, eNOS is degraded in response to oxidant stress (Figure 4A), which is consistent with our previous study (26). There is no significant difference in eNOS expression when DUSP4 was overexpressed in the control endothelial cells. More interesting, under H/R treatment, overexpression of DUSP4 reduces H/R-induced oxidant stress and thus improves the stability and expression of eNOS (Figure 4A).

Subsequently, it is necessary to demonstrate whether the upregulation of eNOS by DUSP4 correlates with increased NO production. In this experiment, DAF-2 was used to determine the extent of NO generated from cells. The fluorescent intensity of adduct of NO and DAF-2 was measured using a Zeiss Axiovert 135 microscope and quantified using ImageJ. Clearly, DUSP4 overexpression promotes NO production (4,595.24 ± 780.73 versus 1,788.76 ± 566.71, *P < 0.05 compared to control group) from cells. H/R treatment does not alter NO generation in endothelial cells. However, overexpression of DUSP4 in cells and H/R treatment enhance NO production (10,501.38 ± 3,503.89 versus 1,747.39 ± 780.73, **P < 0.05 compared to H/R group) and thus provide the beneficial effect against H/R-induced oxidant stress (Figure 4B).

### Quantitative PCR Gene Analysis of DUSP4 Overexpression on the Modulation of NOX4 and eNOS Gene Expression

**Figure 5:**
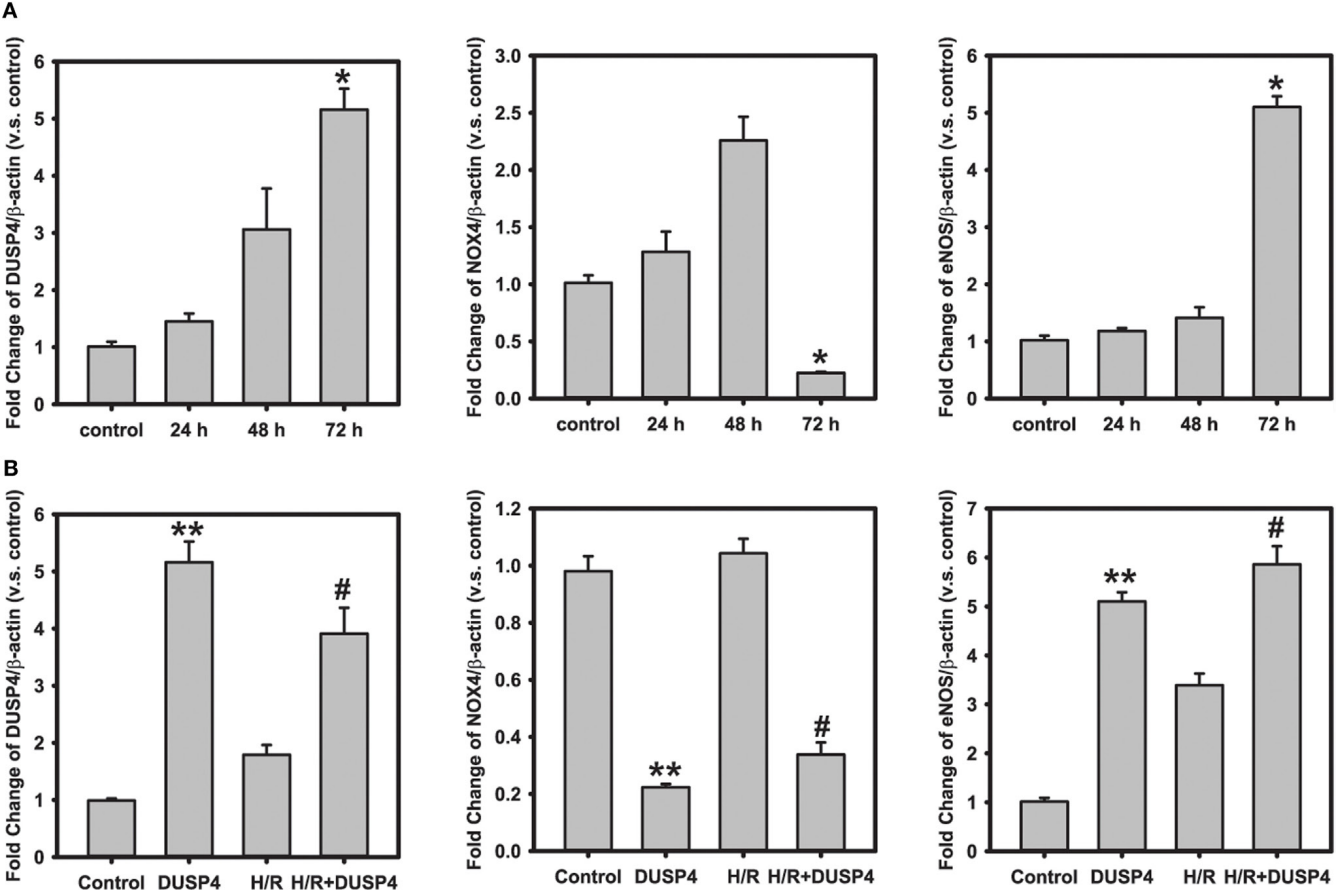
**Quantitative PCR analysis of gene expression**. **(A)** Time course of gene analysis: dual-specificity phosphatase 4 (DUSP4) overexpression downregulates NOX4 gene expression (0.22 ± 0.01 versus control; *P < 0.001) 72 h post-transfection. However, the overexpression of DUSP4 upregulates eNOS expression (5.10 ± 0.19 versus control; *P < 0.001) at the end of 72 h of expression. **(B)** H/R treatment. After 1 h hypoxia and 30 min reoxygenation treatment, DUSP4 overexpression represses NOX4 gene expression (0.34 ± 0.04 versus control), thus reducing the oxidative stress. Upregulation of eNOS expression (5.84 ± 0.37 versus control) by DUSP4 increases NO generation as seen in Figure 4B, thus providing a survival mechanism against H/R-induced oxidant stress. **versus control and ^#^versus H/R; *P* < 0.001. Data are expressed as mean ± SEM, *n* ≥ 3.

To further determine the molecular mechanism and beneficial effects of DUSP4 overexpression against H/R-induced oxidant stress, qPCR was used to analyze gene expression. At the end of 72 h post-transfection, DUSP4 overexpression (5.16 ± 0.37 versus control; *P < 0.001) downregulates NOX4 gene expression (0.22 ± 0.01 versus control; *P < 0.001). However, overexpression of DUSP4 increases eNOS gene expression (5.10 ± 0.19 versus control; *P < 0.001) 72 h post-transfection (Figure 5A). The increase in eNOS mRNA expression is consistent with the increase in eNOS protein expression and NO generation (Figure 4), thus providing a beneficial effect against oxidative stress. After 1 h hypoxia and 30 min reoxygenation treatment, there is no significant change in NOX4 expression in normal cells. However, NOX4 gene expression is repressed in cells with DUSP4 overexpression after H/R (0.34 ± 0.04 versus control; **versus control and ^#^versus H/R *P* < 0.001). Conversely, overexpression of DUSP4 upregulates eNOS gene expression (5.86 ± 0.37 versus control; **versus control and ^#^versus H/R *P* < 0.001), thus increasing NO production as a survival mechanism against oxidative stress (Figure 5B).

## Discussion

The central concept of this study is to investigate the molecular mechanism and beneficial effects of DUSP4 overexpression in endothelial cells against H/R-induced oxidant stress. Previously, we have shown that DUSP4 is degraded after H/R insult in endothelial cells contributing to overactivation of p38 (21). The uncontrolled activation of p38 is the primary factor for cell death and apoptosis. In this study, we demonstrate that overexpression of DUSP4 in endothelial cells protects from H/R-induced apoptosis and cell death. The molecular mechanism and beneficial effects of DUSP4 overexpression against H/R-induced oxidant stress occur *via* the upregulation of eNOS, suppression of ROS generation, and proper modulation of p38 activity. Therefore, results obtained from this study will provide a new direction for the future treatment of ischemic heart disease and vascular dysfunction.

Dual-specificity phosphatase 4 or MKP2 is an inducible nuclear phosphatase whose primary function is to regulate MAPKs (ERK1/2, JNK, and p38) to prevent them from overactivation (12–17, 19). The sustained activation of MAPKs can lead to uncontrolled proliferation and apoptosis. The earlier study suggested that overexpression of DUSP4 in endothelial cells prevented TNF-α-mediated apoptosis *via* the modulation of JNK and NFκB signaling (13). Our recent work demonstrated that DUSP4 is degraded upon H/R insult leading to hyperphosphorylation of p38 and ultimately contributing to cell death and apoptosis (18). In this study, DUSP4 overexpressed in endothelial cells was used to investigate the molecular mechanism of DUSP4 on the modulation of oxidative stress during H/R insult. Indeed, overexpression of DUSP4 in endothelial cells (Figure 1A) protected cells from H/R-induced apoptosis *via* the inhibition of caspase-3 activation (Figure 1B). This result further supported our previous study that upregulation of DUSP4 by NAC protected cells from Cd^2+^-induced oxidant stress and apoptosis (21). Therefore, DUSP4 is a potential therapeutic target for the treatment of oxidant-derived diseases, especially for ischemia heart disease.

Signal transduction events of MAPKs are critical in determining cell fate, including differentiation, proliferation, survival, and apoptosis. In response to a stimulus, the signal amplification of MAPKs is modulated by a series of phosphorylation events. The activation of ERK1/2 is believed to be a pro-survival signal, on the other hand, the activation of p38 and JNK is thought to be a pro-apoptotic signal (30). However, the uncontrolled activation of this signal cascade can lead to catastrophic consequence. Thus, the tight regulation of MAPK activation is required to maintain the proper cell function (31). It has been known that the negative modulation of MAPK signal cascade is conducted by DUSPs. Moreover, in our previous study, we have demonstrated that the hyperphosphorylation of p38 in the absence of DUSP4 in hearts contributes to the poor functional recovery with larger tissue damage after ischemia (18). To further understand the role of DUSP4 on the modulation of MAPK activity under oxidative stress, in this study, we employ DUSP4 overexpression system and H/R cell model. As expected, phosphorylation of p38 is increased in response to H/R insult (Figures 2A,B). The activation of p38 subsequently phosphorylates its downstream target, MK2, at T334. This result is consistent with our previous work of the hyperphosphorylation of p38 in the absence of DUSP4 in response to H/R treatment leading to cell death *via* apoptotic pathway (18). Overexpression of DUSP4 in turn modulates p38 activity preventing overactivation in response to H/R insult. The decrease in phosphorylation of MK2, a downstream target of p38, further supports our hypothesis that DUSP4 is the key phosphatase regulating p38 activity under oxidative stress.

Reactive oxygen species can be beneficial or detrimental to cell function depending on the amount and duration of generation. Under H/R-induced oxidant stress the cellular antioxidant defense system is impaired (32). As a result, it leads to redox imbalance and activation of apoptotic signals, and ultimately contributes to cell death. Our previous study indicated that DUSP4 gene silencing in endothelial cells increased superoxide generation and made it more susceptible to H/R-induced cell death (18). In mouse hearts, DUSP4 gene deletion upregulates Nox4 protein and gene expression. When subjected to *ex vivo* (I/R) injury, DUSP4^−/−^ hearts have poor functional recovery and a larger tissue injury compared to WT hearts (18). Moreover, we have shown previously that increased superoxide generation after H/R insult enhanced the ratio of GSSG/GSH, which in turn can promote protein S-glutathionylation, especially eNOS S-glutathionylation and thus contribute to endothelial dysfunction (7, 23, 26). In this designed study, overexpression of DUSP4 in endothelial cells decreases H/R-induced superoxide generation (Figure 3A), which prevents cellular oxidative stress and subsequently reduces total protein S-glutathionylation (Figure 3B), an indicator for cellular oxidant stress. Quantitative PCR analysis of NOX4 gene transcript suggests that DUSP4 overexpression dramatically decreases NOX4 gene expression prior to and during H/R insult (Figure 5), and in turn reduces cellular ROS generation during reoxygenation. Therefore, DUSP4 is an antioxidant gene and important for maintaining redox balance in cells.

NO, a small molecule generated by NOS, plays an important role in maintaining vascular function, including regulating vascular tone, vascular growth, platelet aggregation, and inflammation (33–35). A decrease in bioavailable NO is a characteristic feature of vascular dysfunction (36–38). Our previous studies demonstrated that eNOS is reversibly S-glutathionylated in the endothelium of the hypertensive rat or endothelial cells with H/R treatment (24, 39). S-glutathionylation of eNOS uncouples the enzyme switching from NO production to superoxide generation and thus increases oxidative stress and endothelial dysfunction (4, 7). This process can be reversed by increasing the reducing environment or by glutaredoxin 1 (7, 26). Our current work shows that overexpression of DUSP4 enhances eNOS protein (Figure 4A) and mRNA expression (Figure 5). The increase in eNOS protein expression in turn promotes NO generation (Figure 4B) and thus protects against H/R-induced oxidant stress. Moreover, DUSP4 overexpression also reduces NOX4 mRNA expression (Figure 5), which decreases oxidative stress in cells and prevents protein oxidation, such as protein S-glutathionylation and increases protein stability under H/R. Therefore, the decrease in ROS generation in DUSP4 overexpressed cells improves the stability of eNOS protein, which enhances NO production providing a survival mechanism against H/R-induced apoptosis.

**Figure 6:**
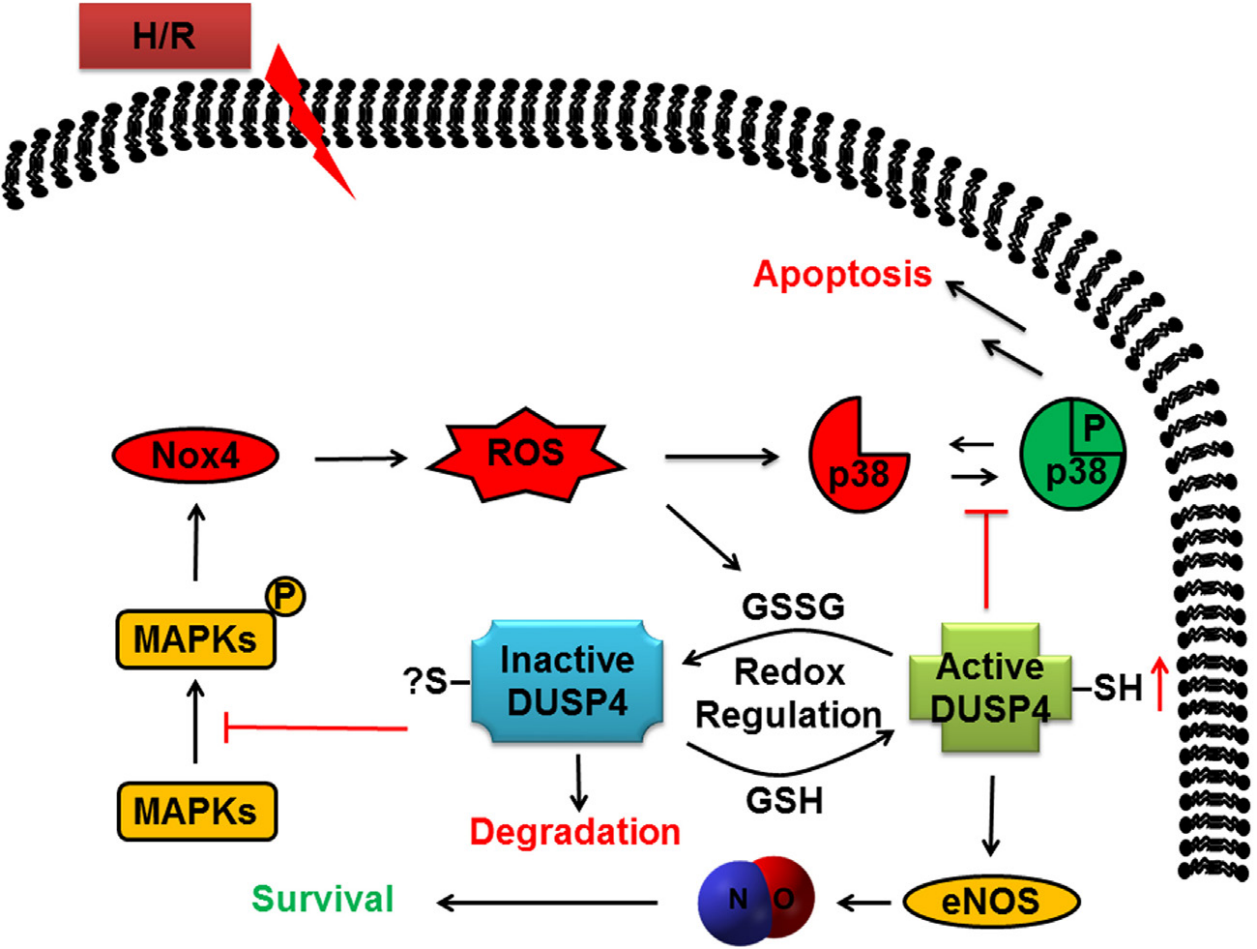
**The role of dual-specificity phosphatase 4 (DUSP4) in response to H/R-induced oxidant stress to determine cell fate**. DUSP4 modulates the activation of mitogen-activated protein kinases in response to H/R injury to influence cellular status toward survival. The cell avoids apoptosis as DUSP4 suppresses uncontrolled activation of p38. Furthermore, DUSP4 overexpression leads to an increase in eNOS expression and stability, which increases cellular NO production to aid the cell in surviving excess reactive oxygen species (ROS). Likewise, overexpression of DUSP4 prompts a decrease in Nox4 expression to cause a subsequent decrease in ROS generation. Overall, DUSP4 acts as an antioxidant gene to provide the cell with multiple mechanisms to protect against H/R-induced oxidant stress.

In conclusion, many therapeutic strategies in the treatment of heart diseases, including ischemic heart disease and vascular dysfunction, aim to improve NO production and at the same time to reduce ROS generation. This study demonstrates that DUSP4 is an excellent therapeutic target for ischemic heart disease and vascular dysfunction. Overexpression of DUSP4 not only upregulates eNOS expression in protein and mRNA levels but also downregulates NOX4 mRNA expression and reduces oxidative stress in response to H/R insult (Figure 6). Therefore, continuing molecular studies in the mechanism of DUSP4 activation in myocardium and endothelium should provide a new direction in the treatment of oxidant-induced cardiovascular dysfunction based on the role of DUSP4 on the modulation of NO production and ROS generation.

## Author Contributions

C-AC and JD, the primary authors, performed most of experiments and data analysis with assistance from MK. JKM performed NO measurement and C-AC and MK performed the confocal microscopy and immunostaining work. MA provided redox biology expertise and guidance. C-AC directed and guided all of the work and prepared the final manuscript with input from all the authors. All authors discussed the results and commented on the manuscript.

## Conflict of Interest Statement

The authors declare that the research was conducted in the absence of any commercial or financial relationships that could be construed as a potential conflict of interest.
